# How does occupational prestige of migrant workers affect farmland transfer in China?

**DOI:** 10.1371/journal.pone.0319468

**Published:** 2025-05-15

**Authors:** Zerong Wang, Jiaxin Fei, Jie Han

**Affiliations:** 1 School of Economics, Zhejiang University of Finance and Economics, Hangzhou, China; 2 School of Business Administration, Southwestern University of Finance and Economics, Chengdu, China; 3 School of Finance, Southwestern University of Finance and Economics, Chengdu, China; Sichuan Agricultural University, CHINA

## Abstract

Occupational prestige, a socioeconomic status metric, has received limited attention in prior studies regarding its influence on farmland transfer. Based on data from the China Family Panel Studies (CFPS) conducted in 2018 and 2020, we analyze the impact of occupational prestige on farmland transfer. The findings reveal that farmers with higher occupational prestige are more likely to transfer their farmland, and the results using the instrumental variable (IV)-Probit model remained significant. Occupational prestige facilitates farmland transfer by enhancing farmers’ awareness of land ownership rights and policies, concurrently diminishing their expectations of land security. Credit-constrained farmers, those with lower income levels, and farmers in the central-western regions of China are more willing to transfer their land. These research insights underscore the significance of fostering inclusive urban employment initiatives and providing upward mobility opportunities for rural migrant workers. Such endeavors are deemed critical for nurturing the development and advancement of China’s rural land rental market.

## 1. Introduction

Land fragmentation poses a significant challenge to agricultural modernization in developing nations, including China. Data from China’s Ministry of Agriculture and Rural Affairs show that the area of family-contracted arable land transferred nationwide was a mere 58 million mu in 2004, By the end of 2020, this figure had risen to 532 million mu, representing a mere 34.09% of the total area of family-contracted arable land. The underutilization and misallocation of land resources have led to sluggish agricultural productivity growth and inefficient economic development across China [1]. Insufficient land circulation and small-scale land holdings are significant barriers to large-scale operations in Chinese agriculture [2]. Therefore, enhancing non-agricultural employment levels and the occupational status of rural laborers holds paramount significance in fostering agricultural-scale operations and promoting sustainable economic development [3].

A large body of literature has examined the factors influencing farmland transfer in China from both macro and micro perspectives. Regarding individual or household micro-level, various characteristics such as age, gender, education level, and physical health conditions of farmers have been found to influence their willingness to transfer farmland [4]. Notably, small-scale farmers who possess an open mindset demonstrate active engagement in the land rental market [5]. Regarding household demographics and mobility constraints, households without elderly members participating in the New Rural Pension Scheme experience reduced marginal effects concerning farmland operation, consequently promoting farmland transfer. This effect becomes more pronounced when household mobility constraints are weaker [6]. Furthermore, studies have revealed that farmers with higher financial literacy are better equipped to address financing constraints and risk-sharing issues, significantly promoting farmland transfer [7]. Turning to macro-level factors, China’s land tenure system, having clarified land property rights, has led to increased land renting by low-productivity farmers [8]. Additionally, it has encouraged the inflow of land by agricultural enterprises and farmer cooperatives, ultimately enhancing the efficiency of the land rental market and facilitating orderly land flow [9–11]. In addition, public interventions by village committees and the development of farmer cooperatives have reduced land transaction disputes between farmland lessors and lessees, thereby facilitating farmland transfer [12,13]. Scholars have also examined the diverse roles of agricultural machinery services in the farmland transfer market [14].

The existing literature closely related to this study primarily explores the interrelationship between off-farm employment and farmland transfer. Off-farm labor market plays a crucial role in promoting the development of the farmland rental market [15]. As urbanization accelerates, a significant number of rural laborers migrate to urban areas for off-farm work, while unutilized land creates conditions for the development of the farmland transfer market [16–19]. Consequently, scholars have scrutinized the off-farm employment behaviors of rural laborers and various associated characteristics [20–22]. Huang et al. [23] found that Chinese farmers engaged in off-farm work are more inclined to transfer farmland. Scholars have also analyzed the underlying mechanisms through which off-farm employment affects farmland transfer, focusing on aspects such as labor division and the differences in production and marketing stages, which lead to reduced working time in primary agriculture activities [24]. The stability of off-farm employment in cities may increase the willingness of migrant workers to stay in urban areas, diminish their reliance on farmland security, and enhance the likelihood of farmland transfer [25]. However, due to the increasing value of farmland, individuals with lower dependence on agricultural production during the urbanization process in China are reluctant to exit farmland contracting rights [26]. Regarding the joint off-farm employment decisions of farming couples, when both the husband and wife engage in off-farm work, it is more probable for land to be rented compared to when only the husband is involved in off-farm work [27]. Furthermore, scholars have emphasized the importance of considering the impact of changes in household population structure on farmland transfer under different types of labor divisions within rural households [6].

Existing research has primarily focused on the correlation between the off-farm employment behavior of rural laborers and farmland transfer, ignoring the social reality that the types of off-farm occupations among migrating labor in rural China exhibit significant differentiation. Different occupational types reflect varying levels of occupational prestige, which may result in differences in farmers’ participation in the farmland market. Occupational prestige refers to the social evaluation of various occupations and serves as an important indicator for measuring economic and social status. It reflects the quantity and quality of social resources possessed. Li [28] identified several key factors that determine individuals’ prestige status in Chinese society, including education, income, power, nature of employment, and engagement in occupations subject to discrimination. Kleinjans et al. [29] also examined the relationship between occupational prestige and gender wage gaps, suggesting that women, compared to men, tend to prefer occupations with higher occupational prestige. The level of occupational prestige influences the occupational choices and aspirations for upward mobility of rural migrant laborers in urban areas. It reflects, to some extent, the disparities in family resources and subsequently affects farmland transfer decisions [30]. The upward occupational mobility of rural laborers leads to variations in occupational types, and the occupational prestige associated with these different types may impact decisions regarding farmland transfer.

Why is it crucial to examine the driving factors of farmland transfer from the perspective of rural laborers’ occupational prestige? First, occupational prestige reflects the economic stability and social recognition of laborers in non-agricultural sectors. Higher occupational prestige is often associated with greater income expectations and job stability. This stability encourages farmers to transfer their land and focus on higher-yielding occupational activities, thereby optimizing the allocation of labor resources. Second, Occupational prestige is not only a driving force behind individual economic behavior but also a reflection of social stratification and occupational mobility trends. Studying the impact of occupational prestige on farmland transfer helps uncover the underlying mechanisms of rural social structure changes and economic transformation. It elucidates how occupational status influences the reallocation of land resources and rural development, providing theoretical support for policies aimed at promoting land market reform and rural revitalization. On this basis, we use data from the China Family Panel Studies (CFPS) conducted in 2018 and 2020 to estimate the effects and mechanisms of farmers’ occupational prestige on farmland transfer.

The contributions of this study are as follows: first, based on the phenomenon of upward occupational mobility among Chinese farmers, we propose a conceptual framework and empirical test on the impact of occupational prestige on farmland transfer. This differs from previous studies that predominantly focused on off-farm employment behavior and characteristics [15,20,21,23,25,31,32]. We highlight the crucial role of occupational prestige differentiation on farmland transfer due to various off-farm occupation types. Second, we further examine the potential mechanisms through which farmers’ occupational prestige influences farmland transfer, specifically exploring the role of land policy acceptance and land security expectations. The research also analyzes the heterogeneity of these effects, recognizing that the impact of occupational prestige differentiation extends beyond factors solely based on off-farm employment behaviors. By investigating these mechanisms, this study provides a more comprehensive understanding of how occupational prestige influences farmland transfer among farmers.

## 2. Conceptual framework

**Fig 1:**
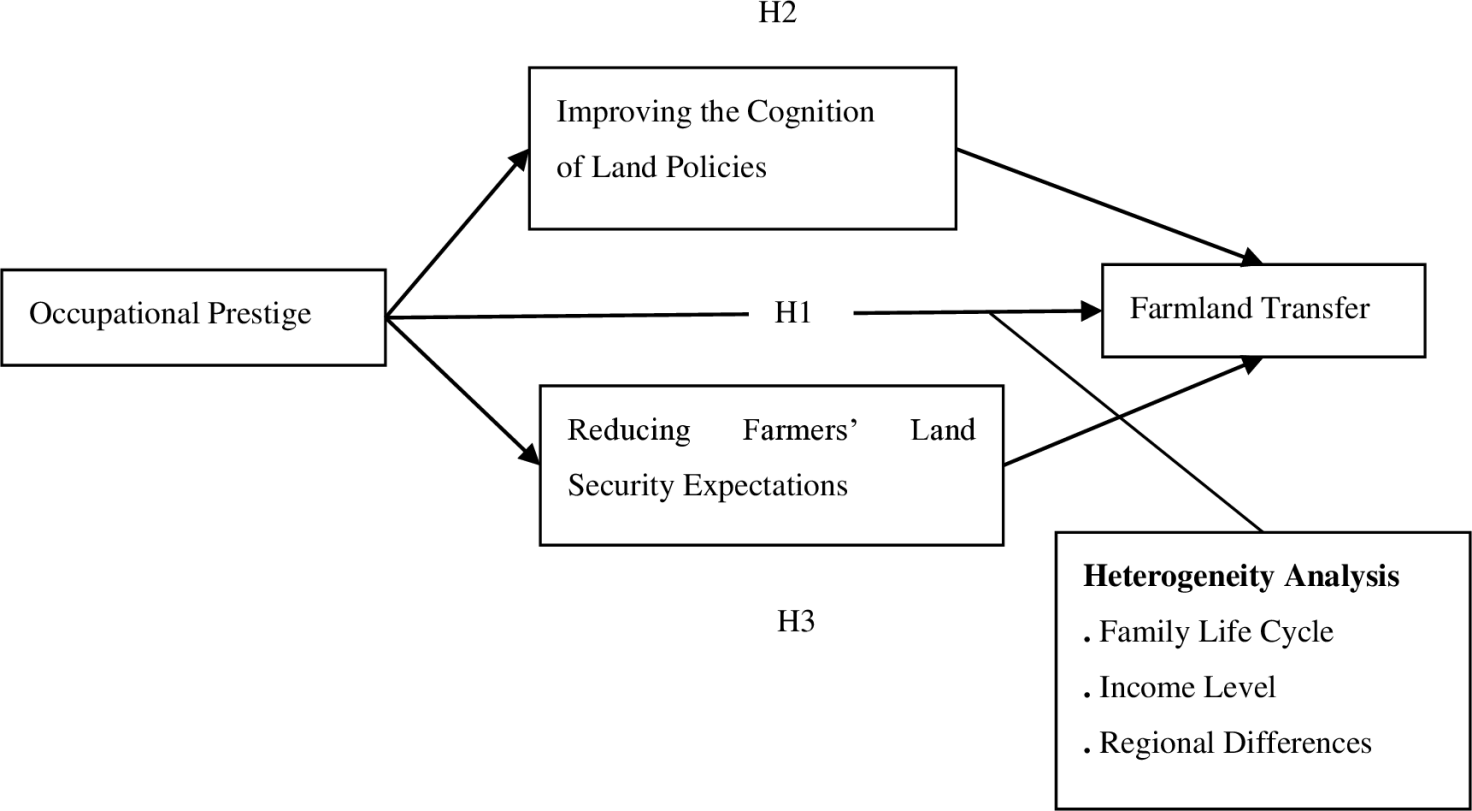
Research framework.

This paper constructs a research framework to examine the impact of occupational prestige on farmland transfer, drawing on “Social Capital Theory” and “Occupational Prestige Theory”. Social Capital Theory emphasizes the role of social networks and trust in shaping individuals’ economic decisions, while Occupational Prestige Theory explains how an individual’s occupational status influences their behavior. The study also explores the mechanisms through two main aspects: the reduction in farmers’ expectations regarding land security and the enhancement of their cognitive abilities. The theoretical framework, as depicted in Fig 1, is further elaborated in the following analysis:

### 2.1. Impact of occupational prestige on farmland transfer

Occupational prestige serves as a reflection of individual judgments and societal evaluations of different occupations, highlighting the variations in resource endowments within the social structure [29]. Different occupational types of rural migrant laborers generate varying levels of occupational prestige. This, in turn, influences farming households’ farmland transfer options. However, it is important to note that farmland holds significant cultural significance in China’s agrarian culture [33–34]. Although the off-farm income of many rural households far surpasses the agricultural production value [26,32,35], the strong “path dependency” of farming culture still imbues land with emotional and mystical value, silently influencing farmers’ decision-making concerning farmland transfer [36]. This cultural influence is particularly prominent among older generations of farmers who are more reluctant to part with their farmland [37]. However, theoretically, whether farmers transfer their farmland is based on rational behavior driven by economic interests and security levels [38]. The influence of occupational prestige on farmland transfer depends on the relative changes in occupational resource endowment compared to land resource endowment.

Factors such as the opportunity cost of agricultural production, the scale of land management, and the development of the farmland transfer market play a role in shaping this influence [39]. Farmers with higher occupational prestige are more likely to evaluate farmland utilization behaviors from a “cost-benefit” perspective, thereby emphasizing economic rationality and exhibiting a higher rate of farmland transfer [40]. Consequently, rural households with higher occupational prestige may reduce their desire to retain farmland and be more willing to withdraw from farmland contractual management rights. Based on the above analysis, the following hypothesis is proposed:

*Hypothesis 1: Occupational prestige has a positive effect on farmland transfer.*

### 2.2. Enhancing awareness of land policies

Occupational prestige positively influences farmland transfer by enhancing cognition of land property rights and policies. On the one hand, farmers’ awareness of land property rights has a positive impact on farmland transfer [17,41,42]. When farmers have a higher level of awareness regarding land property rights, they are more likely to possess land certification and land lease contracts, which enhances the security of their land property rights. This helps to reduce transaction costs and improve transaction efficiency, thereby promoting farmland transfer [43].

Moreover, the occupational prestige associated with different occupation types leads to variations in household characteristics, which subsequently impact farmers’ preferences regarding property rights. Farmers engaged in occupations with higher reputations and greater off-farm income tend to prefer transferring farmland rights [19,23,25], while those with limited off-farm employment opportunities prefer to retain ownership of land use rights, thus hindering farmland transfer [30,34]. On the other hand, the level of farmers’ understanding and judgment of land policies guides their farmland transfer behavior. Factors such as household labor size, social capital, awareness of agricultural business risks, knowledge of land contract law, and judgment of land policy determinants of farmland transfer [4]. Therefore, it is essential to establish platforms for information exchange, enhance farmers’ understanding of land policies, and ensure that farmers make informed decisions during the farmland transfer process [44]. In addition, individual factors, such as farmers’ occupational types, exert varying degrees of influence on their behavioral intentions related to farmland transfer. In summary, farmers with higher occupational prestige demonstrate greater awareness and acceptance of land transfer policies, thus facilitating farmland transfer. Based on these points, the following hypothesis is proposed:

*Hypothesis 2: Occupational prestige promotes farmland transfer by improving farmers’ cognition of land property rights and policies.*

### 2.3. Reducing land security expectations

Occupational prestige has a positive impact on farmland transfer by reducing land security expectations. Firstly, higher occupational prestige is associated with lower income risks and higher income levels [29]. When farmers have steady growth in wage income, it weakens their reliance on land as a source of income and reduces their dependence on land. This, in turn, diminishes the perceived need for land security [45]. Secondly, different occupational types present different occupational prestige levels, which objectively result in differences in farmers’ employment stability preferences and their long-term holding preferences for land assets [46]. Lastly, the willingness to settle in cities for different types of occupations is quite different [25]. Families with higher occupational prestige exhibit greater job satisfaction, which may increase their willingness to migrate long distances and have a stronger desire to settle in urban areas, thereby playing an important role in substituting the land security function [47].

Farming households with higher occupational prestige experience both income growth effects and enhanced employment stability, which reduces expectations of land security. Lower land security expectations have an important role in promoting farmland transfers [48]. On the one hand, the weakening of land security has resulted in a significant shift of the agricultural workforce towards urban areas and a transition from off-farm employment diversification to off-farm employment specialization [37]. Given the increased opportunity cost of engaging in agricultural production, the likelihood of agricultural operation decreases, leading farmers to be more willing to transfer farmland [49]. On the other hand, higher occupational prestige indicates a greater likelihood of farmers entering formal employment in enterprises or institutions, leading to greater social security coverage. Access to corporate benefits and social security reduces farmers’ risk aversion toward their future livelihoods and strengthens their willingness to transfer farmland [15,31].

In conclusion, the higher resource endowment embedded in occupational prestige can directly affect the farmland use process, weaken the land security function, and indirectly influence the decision to transfer farmland. Based on these points, the following hypothesis is proposed:

*Hypothesis 3: Occupational prestige promotes farmland transfer by reducing farmers’ expectations of land security.*

## 3. Research methods

### 3.1. Data

The data were taken from the China Family Panel Studies (CFPS), launched in 2018 and 2020 by the Institute of Social Science Survey of Peking University. This extensive dataset encompasses information from 25 out of 33 provinces within China. The questionnaire mainly centered on questions about individuals, households and communities, reflecting various aspects of social, economic, demographic and educational changes. The article mainly focuses on respondents’ occupations, personal characteristics, household characteristics, subjective cognitive information, and farmland transfer-related information. The respondents’ occupational information, personal characteristics, and subjective perceptions were obtained from the 2018 and 2020 Personal Self-Responding Questionnaire; the household characteristics and farmland transfer status were obtained from the 2018 and 2020 Household Economic Questionnaire. By matching the data from the personal self-responding questionnaire, household economic questionnaire, and household member questionnaire using household codes, we excluded several categories of missing data, such as “unknown”, “refusal to answer”, “not applicable”, “unclear occupational description and unable to classify” and “missing”. A total of 8,089 valid household samples were obtained by eliminating missing data in the categories of “unclear occupation description, unable to classify” and “missing”.

### 3.2. Model and summary statistics

The Probit model is a discrete choice model based on the assumption that the error term follows a normal distribution. The dependent variable in this study is whether farmland is transferred, which is a binary variable. Therefore, we construct a Probit model to study the impact of occupational prestige on farmland transfer.

$Prob\left({Land\_out}_{i}=1\right)=\Phi (\beta_{0}+\beta_{1}{Occpre}_{i}+\beta_{2}X_{i}+\beta_{3}\theta_{j}+\beta_{4}\delta_{t}+\mu )$

The ${Land\_out}_{i}$ indicates whether the respondent has transferred farmland or not (1 = yes; 0 = no). ${Occpre}_{i}$ is the core independent variable, which measures the occupational prestige per worker. $X_{i}$ represents the control variable of individual farmers and families; $\theta_{j}$ is a provincial fixed effect; $\delta_{t}$ is a year fixed effect.

### 3.3. Variables setting

Dependent variable: farmland transfer. Referring to the study by Deng et al. [50], this paper defines farmland transfer based on the question “Do you rent out your farmland?” from CFPS 2018 and 2020. Regardless of whether the household receives rent for the transferred farmland, if the respondent answers “yes”, it is considered farmland transfer behavior and assigned a value of “1”. If the response is “no”, the household is considered not to have transferred farmland and is assigned a value of “0”.

The key explanatory variable is occupational prestige. We utilize the Standard International Occupational Prestige Scale (SIOPS) provided by Ganzeboom and Treiman [51] and assign each rural worker a SIOPS score based on their occupational description in the CFPS data, which reflects the socioeconomic status of different occupations from both subjective and objective perspectives. According to the question “What do you do specifically in this job?” in the CFPS questionnaire, the personal occupational prestige score in the CFPS database is obtained. the CFPS database is used to obtain an individual’s occupational prestige score, which ranges from 1 to 100. Considering that farmland transfer behavior is scored on a household basis, while occupational prestige is scored on an individual basis, this paper calculates the mean occupational prestige of members of the labor force aged 16–60 in each rural family who are employed.

As can be seen in Table 1, the control variables include household head-level characteristics and household-level characteristics. Considering the availability of data and referring to previous studies, the household-level characteristics include age, age squared/100, marital status, education level, and internet usage [25,32,44,52]. The age of the household head and age squared may have a non-linear effect on the farmland transfer; the married head of the household indicates that the socio-economic environment of the household is more complex and makes a difference to the decision to transfer farmland; the ability of the household head to engage in non-farm work is stronger if the household head has a higher education level; the frequent use of the Internet by the household head makes it easier to optimize the information environment, reduce transaction information costs and increase the probability of transferring farmland.

**Table 1:** Variable definition description.

Variables Categories	Variables	Abbrev	Definitions
Explained Variable	Farmland transfer	Farmland	In the past 12 months, have you transferred farmland? yes = 1; no = 0
Core Explanatory Variable	Occupational prestige per work	Occpre	The mean of the occupational prestige of the family workforce
Control Variables	Age	Age	Age of head of household
	Age2	Age2	Age squared/100
	Marriage	Marriage	The head of household is married = 1; otherwise = 0
	Education	Edu	Years of education of the head of household
	Internet use	Internet	Whether the head of household uses a mobile phone to surf the Internet? yes = 1; no = 0
	Household population size	Population	The mean of total household population
	Labor ratio	Rlabor	Labor force/total family population
	Family per capita net income	Capita	The logarithmic value of household per capita net income in the past 12 months
	Total value of household financial assets	Finasset	The mean of total household financial assets
	Land assets	Landasset	Is the land asset value greater than 1,000 yuan? yes = 1; no = 0
	Farmland transfer within the same village	Sametransf	Average probability of land transfer by other households within the same village excluding the household itself

Household-level characteristics include household population size, proportion of household labor force, per capita household net income, total value of household financial assets, and land value [7,18]. Previous studies using CFPS data have shown that rural household behavior is influenced by neighbor-to-neighbor effects, which are referred to as neighborhood effects or spillover effects [53–54]. Therefore, this paper includes the average farmland transfer rate within the same village as a control variable in the model. The description of variables is shown in **Table 2**.

**Table 2:** Descriptive statistics of variables.

Variables	Abbrev	N	Mean	S.D.	Min	Max
Farmland transfer	Farmland	8,089	0.1827	0.3865	0	1
Occupational prestige per work	Occpre	8,089	31.0674	10.1249	19	88
Age	Age	8,089	45.0514	9.4607	16	59
Age2	Age2	8,089	21.1913	8.0064	2.5600	34.8100
Marriage	Marriage	8,089	0.8912	0.3114	0	1
Education	Edu	8,089	1.7191	0.8325	1	6
Internet use	Internet	8,089	0.5732	0.4946	0	1
Household population size	Population	8,089	4.1916	1.8591	1	21
Labor ratio	Rlabor	8,089	0.6534	0.2541	0.0833	1
Family per capita net income	Capita	8,089	9.5520	0.9390	0	15.0094
Total value of household financial assets	Finasset	8,089	7.5941	4.5151	0	15.7614
Land assets	Landasset	8,089	0.7458	0.4354	0	1
Farmland transfer within the same village	Sametransf	8,089	0.0011	0.0037	0	0.0200

### 3.4. Characterization of the occupation

**Fig 2:**
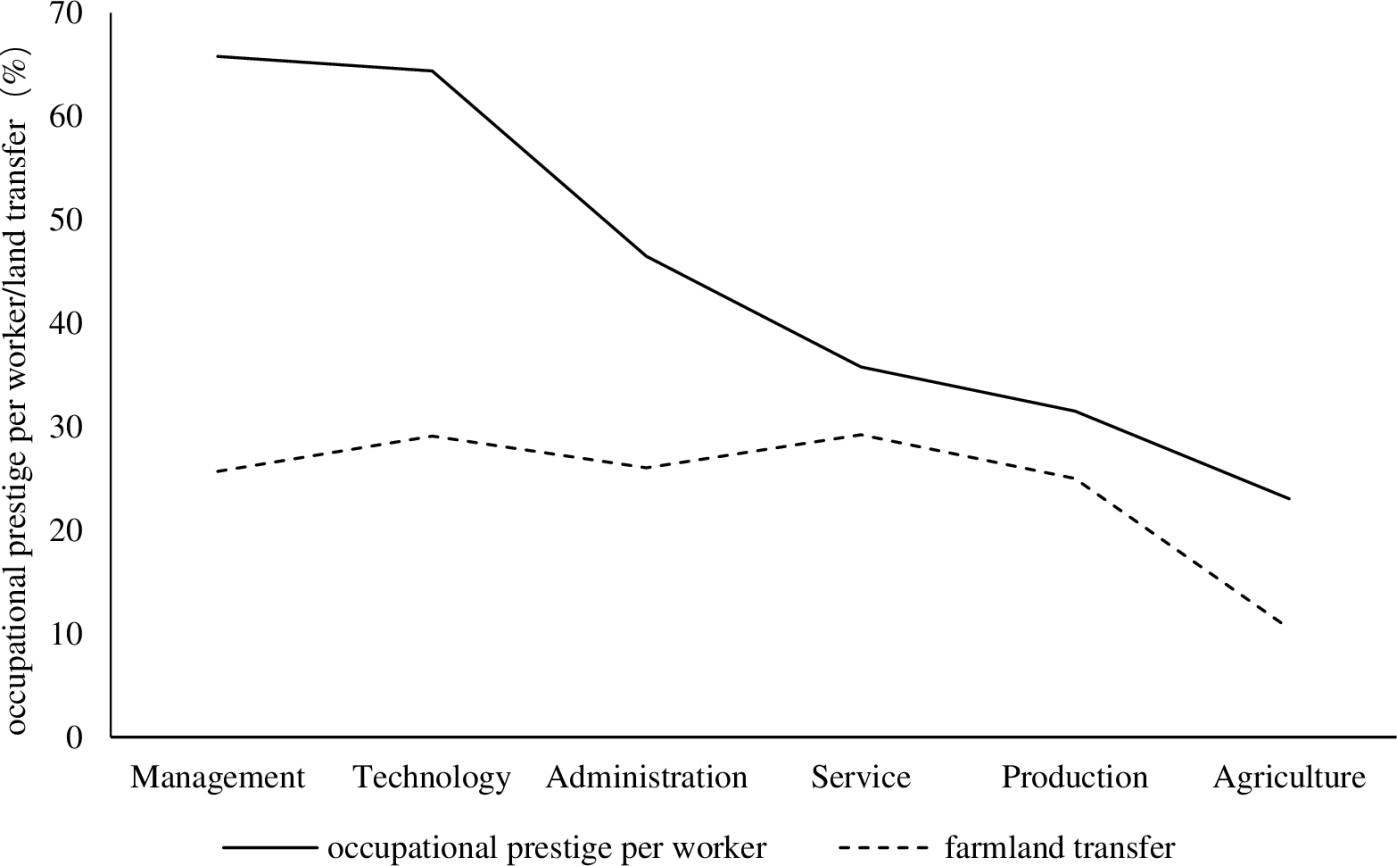
Occupational prestige per worker of different occupational types and farmland transfer.

According to the CFPS data on the job descriptions from 2018 and 2020, occupations are divided into management responsibility, professional technology, administrative affairs, commercial services, production and transportation, and agriculture, forestry, animal husbandry, and fishery categories, respectively. The data excluded individuals with insufficient sample sizes, such as military personnel and other difficult-to-classify occupations. **Fig 2** illustrates the occupational prestige and farmland transfer phenomenon of farmers among different occupational types.

Firstly, there are significant differences in occupational prestige among different occupational categories. The administrative affairs exhibit the highest average occupational prestige, at 65.79. The professional technology categories follow with a slightly lower average of 64.38. The agricultural category has the lowest average occupational prestige.

Secondly, there are large differences in the farmland transfer probability across different occupational types. The commercial services demonstrate the highest farmland transfer probability, reaching 29.22%. Conversely, the agricultural category has the lowest farmland transfer probability, at 10.49%, suggesting a lower inclination for individuals in this category to transfer their farmland.

Lastly, the trends in farmland transfer probabilities align closely with variations in occupational prestige. Specifically, higher occupational prestige is associated with a higher probability of farmland transfer. This observation suggests a positive correlation between occupational prestige and farmland transfer.

### 3.5. The relationship between occupational prestige and farmland transfer

**Table 3** presents the mean difference test for households grouped by high and low occupational prestige. Firstly, the probability of farmland transfer for households with high average occupational prestige is 24.10%, while for households with low average occupational prestige, it is 12.44%. The mean probability of farmland transfer is higher for households with high occupational prestige compared to those with low occupational prestige. Secondly, the mean probability of farmland transfer for households with high head-of-household occupational prestige is 24.04%, significantly higher than the mean probability of farmland transfer for households with low head-of-household occupational prestige, which is 11.92%. The mean difference test results remain significant at the 1% level of significance. Overall, there is a significant positive correlation between occupational prestige and the probability of farmland transfer.

**Table 3:** The relationship between occupational prestige and farmland transfer.

VARIABLES	Farmland Transfer	Mean Difference
High Occupational Prestige Per Worker	24.10	-0.1167***
Low Occupational prestige Per Worker	12.44	
High Householder Occupational Prestige	24.04	-0.1215***
Low Householder Occupational Prestige	11.92	

## 4. Results

### 4.1. Basic result

We use the Probit model to estimate the effect of occupational prestige on farmland transfer, and the regression results are presented in **Table 4**. The marginal effect of average occupational prestige per work (Occpre) is 0.0038 in column (1), indicating that for each unit increase in the occupational prestige index, the probability of farmers engaging in farmland transfer increases by approximately 0.38%. It implies that occupational prestige has a significant positive effect on farmland transfer. This conclusion is consistent with Su et al. [25], which demonstrated that non-agricultural employment stability has a positive impact on farmland leasing. High occupational prestige indicates greater stability, which also significantly promotes farmland transfer. Hypothesis 1 has been confirmed. In addition, the significance and sign of the coefficients of the control variables are generally consistent with the existing literature [32,45].

**Table 4:** The impact of occupational prestige on farmland transfer.

VARIABLES	Farmland Transfer	
	Probit	IV-Probit
	(1)	(2)
Occpre	0.0038^**^	0.0593^***^
	(0.0004)	(0.0134)
Age	0.0003	-0.0154
	(0.0038)	(0.0164)
Age squared/100	0.0019	0.0276
	(0.0045)	(0.0193)
Marriage	-0.0511^***^	-0.2188^***^
	(0.0138)	(0.0570)
Edu	-0.0009	-0.1577^***^
	(0.0057)	(0.0529)
Internet	0.0456^***^	0.01183^***^
	(0.0098)	(0.0439)
Population	-0.0100^***^	-0.0683^***^
	(0.0029)	(0.0138)
Rlabor	-0.0588^***^	-0.3163^***^
	(0.0198)	(0.0848)
Capita	0.0392^***^	0.0388
	(0.0058)	(0.0465)
Finasset	-0.0002	-0.0030
	(0.0010)	(0.0040)
Landasset	0.1212^***^	0.6068^***^
	(0.0115)	(0.0557)
Sametransf	5.0730^***^	13.0655^**^
	(1.0484)	(5.5107)
province fixed effect	Yes	Yes
Year fixed effect	Yes	Yes
Observations	8086	7628
F value		76.00
T value		11.02
Wald test		9.70
		(0.0018)

Note:

***,

**, and

*are significant at the 1%, 5%, and 10% levels, and robust standard errors in parentheses.

### 4.2. Endogenous analysis

The regression results indicate that occupational prestige facilitates farmland transfer by enhancing farmers’ awareness of land policies and reducing their expectations of land security. However, the study may face a reverse causality issue, as farmers who transfer their farmland might become more focused on non-agricultural employment, thereby increasing their occupational prestige, occupational prestige may be an endogenous variable. To test the robustness of the model results, according to the approach of Tan et al. [7], the average occupational prestige of household labor in the same village other than the target household as a tool variable. The study employs the IV-Probit model, which mitigates the reverse effect of current farmland transfer behavior on occupational prestige. The results of the instrumental variable are presented in column (2) of **Table 4**. The p-value of the Wald test is 0.0018, rejecting the null hypothesis at a significance level of 1%. This indicates that occupational prestige is endogenous. Meanwhile, the F-value of 76.00 in the IV-Probit one-stage test is greater than the critical value of 16.38 at the 10% level, indicating that there is no weak instrumental variable. The results of the instrumental variable show a significant positive coefficient for occupational prestige at the 1% level.

### 4.3. Robustness test

#### 4.3.1 Replace the core explanatory variables.

In the base regression model, the age of household occupational prestige is defined as 16–60 years. Considering the deferred retirement and the fact that many farmers over 60 years of age are still working in agriculture or off-farm work, the average household occupational prestige is replaced by the overall household occupational prestige to conduct robustness tests.

**Table 5** shows the results of the Probit and IV-Probit estimates after replacing the range of explanatory variables. The table shows that the instrumental variables are significant at the 1% level of significance, proving that there is still a strong correlation between the two. Meanwhile, the first stage F-value is 77.07 > 16.38, which indicates that the strong instrumental variable is satisfied. The Wald test is 0.0025, rejecting the null hypothesis that the original model is exogenous. The IV-Probit results show that the marginal impact of overall household occupational prestige on farmland transfer is 0.0590, which is generally consistent with the results of the baseline regression model, indicating that the results are robust.

**Table 5:** Replace the core explanatory variables.

VARIABLES	Farmland Transfer	
	Probit	IV-Probit
Family Overall Occupational Prestige	0.0040^***^	0.0590^***^
	(0.0005)	(0.0136)
Control Variables	Yes	Yes
province fixed effect	Yes	Yes
Year fixed effect	Yes	Yes
Observations	8088	7628
F value		77.07
T value		11.49
Wald test		9.15
		(0.0025)

Note:

***,

**, and

*are significant at the 1%, 5%, and 10% levels, and robust standard errors in parentheses.

#### 4.3.2 Winsorize method.

Considering that the empirical results are affected by extreme values, we winsorized the occupational prestige scores of 16–60 years in farm households at the 1st and 99th percentiles. **Table 6** shows that the original model and the instrumental variable regression model are significant at the 1% significance levels respectively, and both regression results demonstrate a significant correlation between occupational prestige and farmland transfer. This is consistent with the results of the baseline regression model.

**Table 6:** Winsorize method.

VARIABLES	Farmland Transfer	
	Probit	IV-Probit
	(1)	(2)
Occpre	0.0041^***^	0.0612^***^
	(0.0005)	(0.0138)
Control Variables	Yes	Yes
province fixed effect	Yes	Yes
Year fixed effect	Yes	Yes
Observations	8086	7632
F value		77.66
T value		11.02
Wald test		9.42
		(0.0021)

Note:

***,

**, and

*are significant at the 1%, 5%, and 10% levels, and robust standard errors in parentheses.

#### 4.3.3 Excluding samples with agricultural farmland rental.

Estimate after excluding samples of farmers who had farmland rental. **Table 7** shows that both the original and instrumental models are significant at the 1% level, indicating that after excluding the sample with farmland rental in the current year, the findings still suggest a significant contribution of occupational prestige to farmland transfer out.

**Table 7:** Excluding samples with farmland rental.

VARIABLES	Farmland Transfer	
	Probit	IV-Probit
	(1)	(2)
Occpre	0.0036^***^	0.0552^***^
	(0.0005)	(0.0164)
Control Variables	Yes	Yes
province fixed effect	Yes	Yes
Year fixed effect	Yes	Yes
Observations	7096	6655
First stage F value		67.21
T value		9.04
Wald test		5.83
		(0.0158)

Note:

***,

**, and

*are significant at the 1%, 5%, and 10% levels, and robust standard errors in parentheses.

## 5. Mechanism test

### 5.1. Improving the cognition of farmland policies

Two proxy variables have been selected to measure the level of land ownership and policy awareness in this paper. The first proxy variable is the average years of education for household members, which is strongly correlated with cognitive abilities. Households with higher levels of education have a higher degree of identification with the “state ownership” attribute of rural farmland, and they are more supportive of central farmland adjustments. In other words, education levels increase the positive perception of farmland property rights and familiarity with land policies, thereby increasing the likelihood of farmland transfer. The second proxy variable is household book collection. The more books a household possesses, the higher its cognitive level. Not only do households with a larger collection of books have a more accurate understanding of land property rights, but they also possess stronger implementation capabilities related to farmland transfer.

We define households with an average education level per labor force member of nine years or less as low-education households and those with more than nine years as high-education households. By introducing the interaction term between occupational prestige and low-education households, the estimation results are in **Table 8**, column (1). The coefficient of the interaction term is significantly positive. This indicates that for low-education households, higher occupational prestige is associated with a higher intention to transfer farmland.

**Table 8:** Mechanism test.

VARIABLES	Farmland Transfer			
	(1)	(2)	(3)	(4)
Occpre	0.0057^*^	0.0129^***^	0.0030	0.0114^***^
	(0.0029)	(0.0021)	(0.0027)	(0.0028)
Occpre × Low Education Level	0.0150^***^			
	(0.0035)			
Low Educational Level	-0.5291^***^			
	(0.1373)			
Occpre × Low Book Collection		0.0085^**^		
		(0.0034)		
Low Book		-0.2070^*^		
		(0.1117)		
Occpre × Working Income is Lower than Agricultural Income			0.0187^***^	
			(0.0034)	
Working Income is Lower than Agricultural Income			0.5457^***^	
			(0.1132)	
Occpre × Low-Level Family Pension				0.0068^**^
				(0.0032)
Low-Level Family Pension				-0.2908^***^
				(0.1087)
Control Variables	Yes	Yes	Yes	Yes
province fixed effect	Yes	Yes	Yes	Yes
Year fixed effect	Yes	Yes	Yes	Yes
Observations	8086	8086	8086	8086

Note:

***,

**, and

*are significant at the 1%, 5%, and 10% levels, and robust standard errors in parentheses.

The sample is divided into low and high-book collection households based on the median household book collection. The interaction term between occupational prestige and low book collection households is introduced in the model for estimation. The results in **Table 8** column (2), show a significant positive coefficient for the interaction term. This indicates that when the household has a lower cognitive ability, as indicated by a lower book collection, households with higher occupational prestige are more likely to transfer farmland compared to those with lower occupational prestige. Occupational prestige has a more significant effect on farmland transfer among households with a limited understanding of land ownership and transfer policies. Thus, mitigating the problem of low farmland transfer efficiency caused by differences in the understanding of farmland ownership and transfer policies. This conclusion is consistent with Wang et al. [42], who found that land tenure security promotes land transfer. This finding further confirms the robustness of the research conclusions. Hence, hypothesis 2 is confirmed.

### 5.2. Reducing farmers’ farmland security expectations

Selection of two proxy variables to define farmland security expectations. The first category is that off-farm income is greater than farm income, indicating low farmland security expectations for households. Referring to the approach of Xu and Lu [35], the relative change between off-farm income and farm income indicates the level of farmland security expectations of households. When off-farm income exceeds farm income, it implies low farmland security expectations, and it is assigned “1”; otherwise assigned “0”. According to CFPS2018 and 2020 data, income from work is the sum of the labor income of rural households in the previous year. Agricultural income is the sum of the value of all farming and forestry products, agricultural subsidies, and reforestation of rural households in the previous year. The second category of proxy variable is represented by remittances from children to rural households, indicating low farmland security expectations. In the process of urbanization, when the children’s family pension model is impacted, farming households will face the pressure of increased liquidity constraints, strengthening the farmland pension security function [6]. Based on the questionnaire, “In the past 12 months, the total amount of money sent home or brought home by all the people working outside your household”, this paper assumes that the non-resident members of farm households send money or bring home money to represent the prominent pattern of children’s retirement, then its alternative function of farmland security will be strengthened, and assigned to “1”, while assigned “0”.

Firstly, the sample was divided into two categories of farm households with high and low farmland security expectations based on the relative changes in off-farm and farm incomes. When off-farm income is greater than farm income it represents a low farmland security expectation for farm households, whereas the opposite indicates households with high farmland security expectations. The interaction term between occupational prestige and off-farm income less than farm income (high farmland security expectations) was introduced into the model for testing. The results are shown in **Table 8**, column (3). The marginal effect of the interaction term is 1.87%, which is significant at the 1% level of significance. This indicates that for households where off-farm income is smaller than farm income, higher occupational prestige has a more significant promoting effect on farmland transfer. In other words, occupational prestige has a more pronounced effect on facilitating farmland transfer among households with high farmland security expectations. These results are consistent with the findings of Wang et al. [34] that the role of cultivated land with contract rights owned by farm households as a means of production is decreasing.

Secondly, using the criterion of whether remittances are sent, households without remittances are classified as having high farmland expectations, while households with remittances are categorized as having low farmland security expectations. To test the interaction effect between occupational prestige and households without remittances (high farmland security expectations), it is included in the model. The results in **Table 8**, column (4), demonstrate a positive effect of the interaction term. This indicates that when non-resident members do not send remittances, the substitution effect of intergenerational support for farmland-based retirement security by children’s households is weakened. Moreover, higher occupational prestige significantly promotes farmland transfer among households with high farmland security expectations. Thus, Hypothesis 3 is supported.

## 6. Heterogeneity analysis

### 6.1. Heterogeneity of the family credit constraint

Farmers’ credit conditions are closely linked to the farmland transfer market [55]. Wang et al. [56] highlight that digital inclusive finance serves as a means to promote the market-oriented transfer of farmland and the efficient utilization of land resources. Therefore, this study aims to extend the analysis by examining whether occupational prestige influences farmland transfer differently for farmers with varying levels of credit constraints.

The questionnaire posed the question: “Have you ever experienced a loan rejection? “ Respondents who reported experiencing a loan rejection were classified as credit-constrained and assigned a value of 1, whereas those who had not faced such rejections were categorized as not credit-constrained and assigned a value of 0. As presented in **Table 9**, for the credit-constrained sample, the coefficient of occupational prestige is 0.016 and significant at the 1% level. In comparison, for the non-credit-constrained sample, the coefficient is 0.015, and significant at the 1% level. These findings indicate that credit-constrained farmers are more likely to transfer farmland as their occupational prestige increases, compared to those without credit constraints. Our results are consistent with Amanullah et al. [57] who suggest that credit constraints can lead to an increase in cultivated land area. Farmers facing credit constraints, due to limited access to capital, are more inclined to transfer their farmland to optimize resource allocation and focus on higher-yield occupational activities. An increase in occupational prestige enhances their economic expectations and social credit, helping to alleviate credit pressure and increasing the likelihood of farmland transfer [58].

**Table 9:** Heterogeneity of family credit constraint.

VARIABLES	Farmland Transfer	
	Credit-constrained	Not Credit-constrained
Occpre	0.0163^***^	0.0154^***^
	(0.0035)	(0.0021)
Control Variables	Yes	Yes
province fixed effect	Yes	Yes
Year fixed effect	Yes	Yes
Observations	2146	5934

Note:

***,

**, and

*are significant at the 1%, 5%, and 10% levels, and robust standard errors in parentheses.

### 6.2. Income heterogeneity

Farm household income may have an impact on the willingness to exit farmland [32]. Therefore, we will examine the impact of occupational prestige on farmland transfer among different income levels.

In this paper, the sample is divided into high-income and low-income groups based on the median household net income. Table 10 shows that compared to the high-income group, occupational prestige has a stronger effect on promoting land transfer among the low-income group. Low-income groups often face greater economic pressures, leading to a more urgent need to increase income and improve their quality of life. In this context, enhanced occupational prestige can provide them with additional economic and social resources, making them more inclined to transfer land in favor of pursuing higher-income occupations or career changes. Our results are consistent with Tan et al. [7], who suggest that total income reduces the likelihood of land transfer.

**Table 10:** Income heterogeneity.

VARIABLES	Farmland Transfer	
	Low-Income	High-Income
Occpre	0.0194^***^	0.0128^***^
	(0.0030)	(0.0023)
Control Variables	Yes	Yes
province fixed effect	Yes	Yes
Year fixed effect	Yes	Yes
Observations	3971	4112

Note:

***,

**, and

*are significant at the 1%, 5%, and 10% levels, and robust standard errors in parentheses.

### 6.3. Regional heterogeneity

Regional differences in agricultural resource endowments and management practices in China have led to distinct patterns of industrial development and variations in occupational prestige within farming households. In terms of industry development, the eastern regions are mainly capital-intensive and technologically advanced agricultural practices. While the Midwest regions focus more on mechanized and large-scale farming. As for the characteristics of occupational prestige, the average occupational prestige per labor unit is highest in the eastern region, with a value of 32.75, followed by the Midwest regions with an average of 30.20. Building upon these observations, we investigate the regional heterogeneity in the impact of occupational prestige on farmland transfer.

We divide the regions into eastern and Midwest regions to estimate the impact of occupational prestige on farmland transfer. The estimated results are shown in Table 11. The coefficient of average occupational prestige per work unit is significant at the 1% level for the eastern region in column (1), the coefficient value is 0.014. The significance at the 1% level for the central-western region in column (2), the coefficient value is 0.016. This implies that, compared to the eastern region, higher occupational prestige among farming households in the central-western region has a greater promoting effect on farmland transfer. On one hand, in the eastern region, farming households with high occupational prestige, as their income levels increase, weaken their social security function of farmland, and strengthen their emotional ownership of farmland. This is consistent with previous research [18], which indicates that as the level of non-farm employment increases, farmers in central and western regions become less dependent on farmland, thereby enhancing the willingness and behavior of rural individuals with higher occupational prestige to transfer their land.

**Table 11:** Regional heterogeneity.

VARIABLES	Farmland Transfer	
	East	Midwest
Occpre	0.0143^***^	0.0163^***^
	(0.0030)	(0.0023)
Control Variables	Yes	Yes
province fixed effect	Yes	Yes
Year fixed effect	Yes	Yes
Observations	2754	5332

Note:

***,

**, and

*are significant at the 1%, 5%, and 10% levels, and robust standard errors in parentheses.

## 7. Discussions and conclusion

### 7.1. Discussions

Based on data from the China Family Panel Studies (CFPS) conducted in 2018 and 2020, we examine the influence of occupational prestige on farmland transfer among farmers. The empirical results reveal that farmers’ participation in the farmland market cannot solely be attributed to off-farm employment behaviors and that occupational prestige also has a significant effect on farmland transfer. Specifically, for each unit increase in the occupational prestige index, the probability of farmers engaging in farmland transfer increases by approximately 0.38%. This implies that farmers with higher occupational prestige are more likely to transfer their farmland. Mechanism tests demonstrate that occupational prestige affects farmland transfer behavior by enhancing farmers’ awareness of land rights, and policies, as well as by reducing their expectations of farmland security. Moreover, this paper reveals that the promotion effect of occupational prestige on farmland transfer is more pronounced among credit-constrained farmers, those with lower income levels, and farmers in the central-western region. This study primarily explores how occupational differences in non-farm employment, from the perspectives of occupation type and occupational status, influence farmland transfer, and complements the existing literature on off-farm employment and farmland transfer [15,20,21,23,25,31,32,52].

### 7.2. Conclusion

This study examines the impact of occupational prestige on farmers’ farmland transfer decisions. Overall, higher occupational prestige increases the likelihood of farmland transfer. Enhancing awareness of land rights and policies while reducing expectations of farmland security are key mechanisms underlying the relationship between occupational prestige and farmland transfer decisions among Chinese migrants. The heterogeneity in the effects of occupational prestige on farmland transfer is prominent. Specifically, the facilitating effect of occupational prestige on farmland transfer is more pronounced among credit-constrained farmers, farmers with lower income levels, and those residing in central and western regions of China.

Policy implication: Promoting off-farm employment opportunities and improving social mobility are key to the development of the rural land rental market in China. Expanding stable, better-paying non-agricultural jobs encourages rural labor migration, which facilitates land transfer and agricultural modernization. Additionally, addressing occupational inequality and enhancing career prospects for migrant workers can motivate rural residents to participate in land rental markets. By creating equal opportunities for upward mobility and reducing income disparities, these measures help optimize labor resources, foster rural development, and promote a more inclusive society.

Due to data limitations, this paper only examines the static effect of occupational prestige and does not explore in depth the differential impact of the dynamic changes in occupational prestige on land transfer. Given the anticipated growth of big data in the future, we aspire to further enrich the understanding of the impact of longitudinal changes in occupational prestige on farmland transfer and investigate the role of social networks in shaping land transfer decisions. Additionally, with the increasing digitalization of rural areas, studying how digital platforms interact with occupational prestige to facilitate land transfers would be valuable in subsequent research.

## Supporting information

S1 Data Supporting Information files. (ZIP)

## References

[1] Li R, Li Q, Lv X, Zhu X. The land rental of Chinese rural households and its welfare effects. China Econ Rev. 2019;54:204–17. doi: 10.1016/j.chieco.2018.11.004

[2] Cheng Y, Chung K. Designing property rights over land in rural China. Econ J. 2017;128(615):2676–710. doi: 10.1111/ecoj.12552

[3] Deininger K, Jin S. The potential of land rental markets in the process of economic development: evidence from China. J Dev Econ. 2005;78(1):241–70. doi: 10.1016/j.jdeveco.2004.08.002

[4] Zhang Y, Halder P, Zhang X, Qu M. Analyzing the deviation between farmers’ Land transfer intention and behavior in China’s impoverished mountainous area: a logistic-ISM model approach. Land Use Policy. 2020;94:104534. doi: 10.1016/j.landusepol.2020.104534

[5] Qian C, Li F, Antonides G, Heerink N, Ma X, Li X. Effect of personality traits on smallholders’ land renting behavior: theory and evidence from the North China Plain. China Econ Rev. 2020;62:101510. doi: 10.1016/j.chieco.2020.101510

[6] Xu D, Guo S, Xie F, Liu S, Cao S. The impact of rural laborer migration and household structure on household land use arrangements in mountainous areas of Sichuan Province, China. Habitat Int. 2017;70:72–80. doi: 10.1016/j.habitatint.2017.10.009

[7] Tan J, Cai D, Han K, Zhou K. Understanding peasant household’s land transfer decision-making: a perspective of financial literacy. Land Use Policy. 2022;119:106189. doi: 10.1016/j.landusepol.2022.106189

[8] Gao X, Shi X, Fang S. Property rights and misallocation: evidence from land certification in China. World Dev. 2021;147:105632. doi: 10.1016/j.worlddev.2021.105632

[9] Cheng W, Xu Y, Zhou N, He Z, Zhang L. How did land titling affect China’s rural land rental market? Size, composition and efficiency. Land Use Policy. 2019;82:609–19. doi: 10.1016/j.landusepol.2018.12.037

[10] Wang Y, Li X, Li W, Tan M. Land titling program and farmland rental market participation in China: evidence from pilot provinces. Land Use Policy. 2018;74:281–90. doi: 10.1016/j.landusepol.2017.07.030

[11] Zhang L, Cao Y, Bai Y. The impact of the land certificated program on the farmland rental market in rural China. J Rural Stud. 2022;93:165–75. doi: 10.1016/j.jrurstud.2019.03.007

[12] Li X, Ito J. An empirical study of land rental development in rural Gansu, China: the role of agricultural cooperatives and transaction costs. Land Use Policy. 2021;109:105621. doi: 10.1016/j.landusepol.2021.105621

[13] Zhang J, Mishra AK, Ma X. Mechanism of Chinese farmers’ land rental participation: the role of invisible markets and public intervention. Food Policy. 2023;117:102453. doi: 10.1016/j.foodpol.2023.102453

[14] Liu Y, Heerink N, Li F, Shi X. Do agricultural machinery services promote village farmland rental markets? Theory and evidence from a case study in the North China plain. Land Use Policy. 2022;122:106388. doi: 10.1016/j.landusepol.2022.106388

[15] Kung JK. Off-farm labor markets and the emergence of land rental markets in rural China. J Comp Econ. 2002;30(2):395–414. doi: 10.1006/jcec.2002.1780

[16] Alasia A, Weersink A, Bollman RD, Cranfield J. Off-farm labour decision of Canadian farm operators: urbanization effects and rural labour market linkages. J Rural Stud. 2009;25(1):12–24. doi: 10.1016/j.jrurstud.2008.04.002

[17] CAO Y, Zou J, Fang X, Wang J, Cao Y, Li G. Effect of land tenure fragmentation on the decision-making and scale of agricultural land transfer in China. Land Use Policy. 2020;99:104996. doi: 10.1016/j.landusepol.2020.104996

[18] Gao J, Song G, Sun X. Does labor migration affect rural land transfer? Evidence from China. Land Use Policy. 2020;99:105096. doi: 10.1016/j.landusepol.2020.105096

[19] Huang K, Cao S, Qing C, Xu D, Liu S. Does labour migration necessarily promote farmers’ land transfer-in?—Empirical evidence from China’s rural panel data. J Rural Stud. 2023;97:534–49. doi: 10.1016/j.jrurstud.2022.12.027

[20] Zhou X, Ma W, Renwick A, Li G. Off-farm work decisions of farm couples and land transfer choices in rural China. Appl Econ. 2020;52(57):6229–47. doi: 10.1080/00036846.2020.1788709

[21] Zhu S, Tian C, Hu Y. Chinese migrant workers’ integration into cities and land transfer amid urban–rural population mobility. Int Rev Econ Finance. 2025;97:103784. doi: 10.1016/j.iref.2024.103784

[22] Fang T, Zhou Y, Wang L, Shi D, Duan X. The impact of multiplex relationships on households’ informal farmland transfer in rural China: a network perspective. J Rural Stud. 2024;112:103419. doi: 10.1016/j.jrurstud.2024.103419

[23] Huang J, Gao L, Rozelle S. The effect of off‐farm employment on the decisions of households to rent out and rent in cultivated land in China. China Agric Econ Rev. 2012;4(1):5–17. doi: 10.1108/17561371211196748

[24] Su W, Eriksson T, Zhang L, Bai Y. Off-farm employment and time allocation in on-farm work in rural China from gender perspective. China Econ Rev. 2016;41:34–45. doi: 10.1016/j.chieco.2016.08.006

[25] Su B, Li Y, Li L, Wang Y. How does nonfarm employment stability influence farmers’ farmland transfer decisions? Implications for China’s land use policy. Land Use Policy. 2018;74:66–72. doi: 10.1016/j.landusepol.2017.09.053

[26] Dong H, Zhang Y, Sun Y, Jiang T. To keep or not to keep the farmland? Incentives and barriers to farmers’ decisions in urbanizing China. Habitat Int. 2022;130:102693. doi: 10.1016/j.habitatint.2022.102693

[27] Zhou JK, Wang WB, Gong MY, Huang ZX. Land transfer, occupational stratification and poverty reduction effects. Econ Res. 2020;55:155–71.

[28] Li CL. Prestige stratification in contemporary Chinese society——a measure of occupational prestige and socioeconomic status indices. Sociol Res. 2005;74–102. (In Chinese).

[29] Kleinjans KJ, Krassel KF, Dukes A. Occupational prestige and the gender wage gap. Kyklos. 2017;70(4):565–93. doi: 10.1111/kykl.12149

[30] Liu Z, Rommel J, Feng S, Hanisch M. Can land transfer through land cooperatives foster off-farm employment in China? China Econ Rev. 2017;45:35–44. doi: 10.1016/j.chieco.2017.06.002

[31] Xu D, Deng X, Huang K, Liu Y, Yong Z, Liu S. Relationships between labor migration and cropland abandonment in rural China from the perspective of village types. Land Use Policy. 2019;88:104164. doi: 10.1016/j.landusepol.2019.104164

[32] Xu M, Chen C, Xie J. Off-farm employment, farmland transfer and agricultural investment behavior: a study of joint decision-making among North China Plain farmers. J Asian Econ. 2024;95:101839. doi: 10.1016/j.asieco.2024.101839

[33] Gutiérrez Rodríguez L, Hogarth NJ, Zhou W, Xie C, Zhang K, Putzel L. China’s conversion of cropland to forest program: a systematic review of the environmental and socioeconomic effects. Environ Evid. 2016;5(1). doi: 10.1186/s13750-016-0071-x

[34] Wang Y, Li X, He H, Xin L, Tan M. How reliable are cultivated land assets as social security for Chinese farmers?. Land Use Policy. 2020;90:104318. doi: 10.1016/j.landusepol.2019.104318

[35] Xu Q, Lu Y. Off-farm employment, the social security function of land, and land transfer. Chin Popul Sci. 2018;188:30–41.

[36] Kan K. Creating land markets for rural revitalization: land transfer, property rights and gentrification in China. J Rural Stud. 2021;81:68–77. doi: 10.1016/j.jrurstud.2020.08.006

[37] Song M, Wu Y, Chen L. Does the land titling program promote rural housing land transfer in China? Evidence from household surveys in Hubei Province. Land Use Policy. 2020;97:104701. doi: 10.1016/j.landusepol.2020.104701

[38] de Janvry A, Emerick K, Gonzalez-Navarro M, Sadoulet E. Delinking land rights from land use: certification and migration in Mexico. Am Econ Rev. 2015;105(10):3125–49. doi: 10.1257/aer.20130853

[39] Strijker D. Marginal lands in Europe—causes of decline. Basic Appl Ecol. 2005;6(2):99–106. doi: 10.1016/j.baae.2005.01.001

[40] Zhang Y, Li X, Song W, Zhai L. Land abandonment under rural restructuring in China explained from a cost-benefit perspective. J Rural Stud. 2016;47:524–32. doi: 10.1016/j.jrurstud.2016.06.019

[41] Gao L, Sun D, Huang J. Impact of land tenure policy on agricultural investments in China: evidence from a panel data study. China Econ Rev. 2017;45:244–52. doi: 10.1016/j.chieco.2017.07.005

[42] Wang H, Riedinger J, Jin S. Land documents, tenure security and land rental development: panel evidence from China. China Econ Rev. 2015;36:220–35. doi: 10.1016/j.chieco.2015.09.005

[43] Deininger K, Zegarra E, Lavadenz I. Determinants and impacts of rural land market activity: evidence from Nicaragua. World Dev. 2003;31(8):1385–404. doi: 10.1016/s0305-750x(03)00101-3

[44] Deng X, Xu D, Zeng M, Qi Y. Does Internet use help reduce rural cropland abandonment? Evidence from China. Land Use Policy. 2019;89:104243. doi: 10.1016/j.landusepol.2019.104243

[45] Rao F, Spoor M, Ma X, Shi X. Perceived land tenure security in rural Xinjiang, China: the role of official land documents and trust. China Econ Rev. 2020;60:101038. doi: 10.1016/j.chieco.2017.03.009

[46] Knight J, Yueh L. Job mobility of residents and migrants in urban China. J Comp Econ. 2004;32(4):637–60. doi: 10.1016/j.jce.2004.07.004

[47] Robinson C. Occupational mobility, occupation distance, and specific human capital. J Human Resources. 2018;53(2):513–51. doi: 10.3368/jhr.53.2.0814-6556r2

[48] Lai Z, Chen M, Liu T. Changes in and prospects for cultivated land use since the reform and opening up in China. Land Use Policy. 2020;97:104781. doi: 10.1016/j.landusepol.2020.104781

[49] Brewer J, Larsen A, Noack F. The land use consequences of rural to urban migration*. Am J Agri Econ. 2022;106(1):177–205. doi: 10.1111/ajae.12369

[50] Deng X, Xu D, Zeng M, Qi Y. Does early-life famine experience impact rural land transfer? Evidence from China. Land Use Policy. 2019;81:58–67. doi: 10.1016/j.landusepol.2018.10.042

[51] Ganzeboom HBG, Treiman DJ. Internationally comparable measures of occupational status for the 1988 international standard classification of occupations. Soc Sci Res. 1996;25(3):201–39. doi: 10.1006/ssre.1996.0010

[52] Lu C, Wu A. The impact of migration characteristics on rural migrant households’ farmland use arrangements in China. PLoS One. 2022;17(8):e0273624. doi: 10.1371/journal.pone.0273624 36037159 PMC9423677

[53] Zhao C, Qu X. Peer effects in pension decision-making: evidence from China’s new rural pension scheme. Labour Econ. 2021;69:101978. doi: 10.1016/j.labeco.2021.101978

[54] Gao J, Zhao R, Lyu X. Is there herd effect in farmers’ land transfer behavior? Land. 2022;11(12):2191. doi: 10.3390/land11122191

[55] Porgo M, Kuwornu JKM, Zahonogo P, Jatoe JBD, Egyir IS. Credit constraints and cropland allocation decisions in rural Burkina Faso. Land Use Policy. 2018;70:666–74. doi: 10.1016/j.landusepol.2017.10.053

[56] Wang Y, Zhao Z, Lu C. Does digital inclusive finance increase land rent? Evidence from rural China. Econ Anal Policy. 2024;82:1025–43. doi: 10.1016/j.eap.2024.04.032

[57] Lakhan GR, Channa SA, Magsi H, Koondher MA, Wang J, et al. Credit constraints and rural farmers’ welfare in an agrarian economy. Heliyon. 2020;6(10):e05252. doi: 10.1016/j.heliyon.2020.e05252 33088980 PMC7567925

[58] Chen C, Liu B, Wang Z. Can land transfer relax credit constraints? Evidence from China. Econ Modell. 2023;122:106248. doi: 10.1016/j.econmod.2023.106248

